# Finding a home for hyoliths

**DOI:** 10.1093/nsr/nwz194

**Published:** 2019-11-27

**Authors:** Martin R Smith

**Affiliations:** 1 Department of Earth Sciences, Durham University, UK; 2 Reviewer of NSR

Many of the animal lineages that arose during the Cambrian evolutionary radiation, 540 million years ago, are difficult to relate to living taxa. Constraining the phylogenetic position of these highly disparate taxa allows their idiosyncratic morphologies to illuminate the stepwise establishment of modern body plans [1–3].

Hyoliths are one such lineage; their operculate conical shells are common Palaeozoic fossils. Hyoliths have recently been identified as brachiopods based on the description of an attachment stalk [4] and a tentaculate feeding apparatus [5]. Liu *et al.* [6] present an alternative view, interpreting the putative pedicle as a damaged shell apex, and questioning whether the feeding apparatus ought to be termed a lophophore.

**Figure 1:**
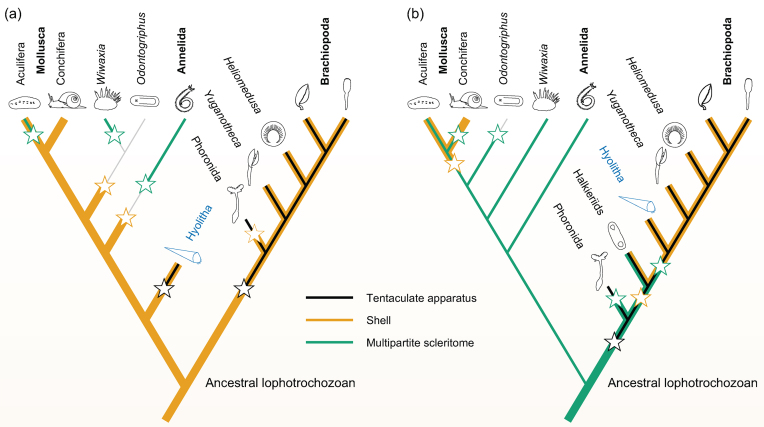
**Figure 1.** Evolutionary implications of interpreting hyoliths as (a) ‘basal lophotrochozoans’ [6]; (b) stem-group brachiopods [4]. Stars denote gains and losses of key attributes. Phylogenetic framework in (a) is a best attempt to accommodate hyoliths close to the common ancestor of Lophotrochozoa.

As hyolith shells lack any opening through which a pedicle might emerge, the description of an attachment stalk in the Chengjiang hyolith *Pedunculotheca* [4] was unexpected. Liu *et al.* [6] demonstrate that this structure lacks the iron enrichment that commonly characterizes Chengjiang pedicles, and propose that an internal mould of the apical shell might come to resemble a pedicle. Even if external ornamentation and a holdfast-like structure are difficult to accommodate in this model, the status of the putative hyolith ‘pedicle’ can no longer be considered secure.

Secondly, Liu *et al*. document a tentaculate feeding apparatus in *Triplicatella*, a member of the basal ‘orthothecid’ grade of hyoliths (unless its ‘arcuate processes’ represent clavicles, a hyolithid characteristic). Considered alongside the derived hyolithid *Haplophrentis* [5], *Triplicatella* helps to calibrate the degree of variation in hyolith feeding structures. Within the limitations of preservational fidelity, the similarities with the *Haplophrentis* apparatus are more obvious than the differences—particularly if the ‘anterolateral arms’ are interpreted as the most lateral of a series of regularly spaced tentacles.

Liu *et al.* [6] consider feeding apparatus morphology to exclude hyoliths from brachiopods, instead proposing a position somewhere at the base of Lophotrochozoa, the clade embracing molluscs, annelids, nemerteans and lophophorates. Similarities between certain hyolith and mollusc shell microstructures are taken to denote a single evolutionary origin (following [7], but despite the diverse and frequently convergent suite of microstructures within hyoliths and molluscs [8,9]).

This proposal has far-reaching evolutionary implications (Fig. 1a). It implies that the ancestral lophotrochozoan bore a shell, rather than a multipartite scleritome [10]—elevating the reliability of the Cambrian fossil record [11] as a chronicle of early Lophotrochozoan evolution. As a lophotrochozoan symplesiomorphy, a shell would be secondarily lost among annelids, in contrast to previous accounts of their early evolution [12, 13]; and homology between the shells of hyoliths, molluscs and brachiopods would prompt a re-evaluation of non-mineralized stem-group molluscs [14].

On the other hand, the similarity between the tentaculate feeding structures of hyoliths and *Yuganotheca* is exactly consistent with the progressive increase in resemblance to the modern-day lophophore that is expected as one ascends, via *Heliomedusa*, the brachiopod stem lineage [15] (Fig. 1b). Even if hyoliths (like craniid brachiopods) lack a pedicle, their bilaterally symmetrical paired valves are comfortably (and parsimoniously [4]) interpreted as homologous with those of *Yuganotheca* and, ultimately, the brachiopods.
